# Contrast-Enhanced Harmonic Endoscopic Ultrasonography in Pancreatic Diseases

**DOI:** 10.1155/2012/786239

**Published:** 2012-11-01

**Authors:** Can Xu, Zhaoshen Li, Michael Wallace

**Affiliations:** ^1^Division of Gastroenterology and Hepatology, Mayo Clinic, Jacksonville, FL 32224, USA; ^2^Department of Gastroenterology, Changhai Hospital, Second Military Medical University, Shanghai 200433, China

## Abstract

Endoscopic ultrasonography (EUS) is the most sensitive imaging method for diagnosis of pancreatic tumors. However, it still has limits in the differentiation between pancreatic cancers and inflammatory tumor-like masses. A novel technology, contrast-enhanced harmonic EUS (CH-EUS), has been developed recently. It can visualize both parenchymal perfusion and microvasculature in pancreas without Doppler-related artifacts. Therefore, it is superior to EUS and CT in detecting small pancreatic masses and differential diagnosis of pancreatic masses. CH-EUS could be used for adequate sampling of pancreatic tumors and may predict the pathological features of the pancreatic solid lesions but still cannot replace EUS-FNA now.

## 1. Introduction

Pancreatic cancer is one of the most devastating diseases with long-term survival being still rare. Therefore, there is an urgent need to develop a method for diagnosing pancreatic cancer at an early curable stage. Endoscopic ultrasound (EUS) is considered to be the most sensitive technology in detecting small pancreatic tumors [1, 2]. However, the differentiation between pancreatic tumors and inflammatory tumor-like masses still remains difficult. The evaluation of vascularity using ultrasound contrast may assist the differentiation of cancers from benign tumors. 

Contrast-enhanced harmonic endoscopic ultrasonography (CH-EUS) is a novel technology which observes both parenchymal perfusion and microvasculature in the pancreas and has been reported to improve characterization of pancreatic cancers from other pancreatic diseases [3, 4]. 

In this paper, we will describe the development of a new technology CH-EUS and will review the advantages and its value in clinical practice in pancreatic diseases [5].

## 2. Development of CH-EUS 

Power-Doppler and color-Doppler have been used for contrast enhanced transabdominal US (CE-US) until recently. Although the contrast agent selectively enhances the useful signal, the main disadvantage of these techniques is the presence the inevitable artifacts such as blooming and overpainting [3]. 

CE harmonic US (CH-US) is a technique that is able to detect signals from microbubbles in vessels with very slow flow without Doppler-related artifacts [5]. 

CH-US is used to characterize tumor vascularity in liver and pancreas. It helps to differential diagnose of benign and malignant liver tumors mainly because of the dual blood supply of the liver via the arterial supply and liver-specific portal vessels. In contrast to liver, pancreas does not contain a dual vascular system. But, several studies showed promising results in differentiating malignant from benign pancreatic lesions in analyzing vascularity with CH-US [6, 7]. 

Recently, contrast-enhanced EUS with Doppler mode (CE-EUS) employing ultrasound contrast agents, which indicate vascularization in pancreatic lesions, has been found to be useful in the differential diagnosis of pancreatic tumors, especially small pancreatic tumors. Sakamoto et al. [8] compared CE-CT and CE-EUS by power Doppler mode using sonographic contrast agent Levovist for detection and differential diagnosis of pancreatic tumors in 156 consecutive patients with suspected pancreatic tumors. Thirty-six of 156 patients examined had tumors of ≤2 cm. The results showed that EUS had significantly higher sensitivity (94.4%) for detection of pancreatic carcinomas of 2 cm or less in comparison to CE-CT (50%). For small pancreatic tumor of ≤2 cm, sensitivities for differentiating ductal carcinomas from other tumors were 50.0%, 11.0%, and 83.3% for CE-CT, Power Doppler or color Doppler mode EUS (PD-EUS) and CE-EUS. CE-EUS was significantly more sensitive than PD-EUS and CE-CT. CE-EUSs are more sensitive than CE-CT in the detection and the differentiation of small pancreatic tumors.

However, just like CE-US, CE-EUS has a couple of limitations such as blooming artifacts, poor spatial resolution, and low sensitivity to slow flow. Consequently, contrast-enhanced harmonic EUS (CH-EUS) with a second-generation ultrasound contrast agent was developed recently. The CH-EUS technique is expected to improve the differential diagnosis of pancreatic disease in the future [9]. 

In 2005, Dietrich et al. [10] first described this new method with a preliminary prototype in six patients by injection of the second generation of contrast agent Sonovue which is composed of microbubbles of sulfur hexafluoride. The technique was subsequently improved by Kitano and his study group in 2008 [11]. They reported their study with a dedicated contrast harmonic method by using a prototype echoendoscope with a broad-band transducer with Sonovue in patients with pancreatobiliary diseases, gastrointestinal stromal tumors, and lymph node metastases. This feasibility study showed that an optical mechanical index of 0.4 allowed successful visualization of parenchymal perfusion and microvasculature of the pancreas during real-time imaging. However, contrast-enhanced power-Doppler EUS (CE-EUS) failed to depict images of the fine vessels and parenchymal perfusion, whereas blooming artifacts of large vessels were observed. These indicated that the contrast harmonic method was superior to the CE-EUS and it could improve the accuracy in evaluate tissue vasculature with EUS imaging. 

## 3. Endoscope Scope and Contrast Agents for CH-EUS

The CH-EUS endoscope has a broad-band transducer and a specified imaging mode. An echoendoscope which is developed for CH-EUS (GF-UCT260, Olympus medical systems, Tokyo, Japan) is now commercially available. The ultrasound image processor for CH-EUS is the Aloka ProSound SSD *α*-10 (Aloka, Tokyo, Japan). CH-EUS technology can detect signals from microbubbles in vessels with a very slow flow without Doppler-related artifacts and can be used to characterize parenchyma perfusion and the vascular structures of pancreatic lesions. Contrast agents are made of gas-filled microbubbles encapsuled by a phospholipid or albumin shell. They are categorized into first and second generation based on the capability for transpulmonary passage and the half-life in the human body. Sonovue and Sonazoid are the most commonly used second-generation contrast agents which produce harmonic signals at low acoustic powers and thus are suitable for EUS imaging at low acoustic powers. Sonovue (Bracco imaging, Milan, Italy) is composed of microbubbles of sulfur hexafluoride within a phospholipid membrane. Sonazoid (Daiichi-Sankyo, Tokyo, Japan; GE Healthcare Milwaukee,WI) consists of perfluorobutane microbubbles with a diameter of 2-3 *μ*m surrounded by a lipid membrane [5]. The clinically used contrast agents are safe and there were no severe long-lasting adverse effects observed.

## 4. CH-EUS in Pancreatic Diseases

Pancreatic solid masses are characterized according to the vascular patterns observed on CH-EUS images. The different lesions have different specialized vascular patterns (Table 1).

## 5. Is CH-EUS Superior to CT or MRI? 

Computed tomography (CT) and magnetic resonance imaging (MRI) are also widely used in pancreatic diseases. Comparing with CT and MRI, EUS has been reported to have higher sensitivity and specificity in the diagnosis of pancreatic tumors for the ability to place the EUS transducer in direct proximity to the pancreas enables accurate preoperative staging of cancer [12].

Kitano et al. [2] reported that CH-EUS was superior to multidetector computed tomography (MDCT) in diagnosing small (≤2 cm) pancreatic carcinomas (*P* < 0.05). In all 12 neoplasms that MDCT failed to detect, 7 ductal carcinomas and 2 neuroendocrine tumors had hypoenhancement and hyperenhancement on CH-EUS, respectively. However, CH-EUS and MDCT did not differ significantly in diagnostic ability with regard to all pancreatic lesions. Michiko et al. [13] compared CH-EUS using Sonazoid as contrast agent with CT and MRI; he demonstrated that the vascular image on CH-EUS was more precise than CT and MRI. CH-EUS may become a new investigation of tumor vasculature, especially for evaluation of pancreatic mass lesions. Intraductal papillary mucinous neoplasms (IPMNs) are a unique entity of pancreatic tumor with a wide spectrum of histological differentiation from hyperplasia to invasive carcinoma. Ohno et al. [14] reported that only CE-EUS revealed mural nodules in 3 (27.3%) of the 11 patients with malignant IPMN treated with resection which were not detected by CT or MRI. Form all the above-reported results, we can see that CH-EUS is superior to CT and MRI scan in diagnosis of pancreatic tumors with higher sensitivity and specificity.

## 6. Is CH-EUS Better Than Conventional EUS?

CH-EUS is an EUS system with a broad-band transducer which enabled the visualization of microvessels and the parenchymal perfusion of the pancreas. It has been shown that most pancreatic cancers exhibit hypovascular heterogeneous enhancement with irregular network-like microvessels. Moreover, it can diagnose pancreatic cancers with a high sensitivity (89–92%) [15]. The same group also reported that CH-EUS is much better than conventional EUS in clearly depicting the outline of six pancreatic carcinomas [2]. A quantitative CH-EUS study showed that the size of the pancreatic mass was assessed significantly effectively by CH-EUS compared with conventional EUS [16]. Recently, Fusaroli et al. [17] reported that CH-EUS could overcome some of the limitations of the conventional EUS, such as confounding factors, biliary stents, and chronic pancreatitis, and could improve the diagnostic accuracy. In their study CH-EUS detected small hypoenhancing lesions clearly in 7 patients who had uncertain standard EUS findings because of biliary stents (*n* = 5) or diffuse chronic pancreatitis (*n* = 2). The final diagnosis was adenocarcinoma in all these patients. In this respect, CH-EUS provided an increase in diagnostic yield of pancreatic adenocarcinoma of almost 8%. Hypovascularity as a sign of malignancy in CH-EUS obtained 92% sensitivity and 100% specificity. A recent pilot study showed that the rated as undecided/indeterminate with EUS and CH-EUS was 13.3% versus 3.3% (*P* = 0.35), and CH-EUS adds minimal imaging time and is accurate, with small improvement over EUS [18].

EUS is probably the most useful method available for evaluating pancreatic cystic lesions, particularly intraductal papillary mucinous neoplasms (IPMN) [19]. Diagnosis of IPMN by EUS depends on whether a mural nodules are detected; moreover, the mural nodule including the size observed by EUS was a reliable preoperative diagnostic finding capable of distinguishing low-risk and high-risk IPMN [20]. However, it is sometimes very difficult to discriminate sludge or mucous clots from mural nodules by conventional EUS. Results showed that CH-EUS could exclude avascular regions (mucous clots), whereas it depicts the mural nodules with enhancement. Therefore, CH-EUS might improve the ability of EUS in depicting mural nodules of IPMN [11, 15].

## 7. Could CH-EUS Aid the Differential Diagnosis of Pancreatic Diseases?

Distinguishing pancreatic adenocarcinoma from other pancreatic masses remains challenging with current imaging techniques. So several study aimed to evaluate the accuracy of CH-EUS in differentiate diagnosis for pancreatic masses. Napoleon's pilot study [21] revealed that of all 18 lesions with a hypointense signal on CH-EUS, 16 were adenocarcinomas. The sensitivity, specificity, negative predictive value (NPV), positive predictive value (PPV), and accuracy of hypointensity for diagnosing pancreatic adenocarcinoma were 89%, 88%, 88%, 89%, and 88.5%, compared with corresponding values of 72%, 100%, 77%, 100%, and 86% for EUS-FNA. Therefore, they concluded that CH-EUS with the new Olympus prototype device successfully visualized the microvascular pattern in pancreatic solid lesions, and might be useful for distinguishing adenocarcinomas from other pancreatic masses.

The majority of cases of both pancreatic cancer and chronic pancreatitis were hypoenhanced and visual discrimination was not possible. A study about CH-EUS for the quantitative assessment of uptake after contrast injection showed that it can aid differentiation between benign and malignant masses. The therapeutic strategy has not been changed after contrast medium injection during CH-EUS [16]. Though the application of CH-EUS in the differential diagnosis of chronic pancreatitis and pancreatic cancer is promising, it still cannot replace the role of EUS-FNA nowadays.

## 8. Could CH-EUS Help Preoperative T-Staging?

In pancreaticobiliary cancers, preoperative T-staging is of great importance to guide the appropriate treatment. EUS has been widely used for local T-staging of pancreaticobiliary cancers. The accuracy of EUS for tumor staging has been shown in a lot of studies to be significantly better than that of any other imaging modalities. However, the precise preoperative T-staging still remains a challenge.

A Japanese study group compared the T-staging accuracy of CH-EUS with use of Sonazoid and conventional harmonic EUS (H-EUS). The results showed H-EUS misstaged the depth of invasion to pancreas (T3) as T2 in a case of ampulla cancer, and misdiagnosed T2 stage of gallbladder cancer as adenomyomatosis (T0) localized in the surface of gallbladder wall. Moreover, H-EUS overstaged two cases of pancreatic carcinoma (T2) and two cases of extrahepatic bile duct carcinoma (T3) without portal vein invasion as T3 and T4 with portal vein invasion, respectively. However, CH-EUS correctly staged all these 6 cases. The overall T-staging accuracy of CH-EUS was significantly higher than H-EUS at 92.4% (24/26) and 69.2% (18/26), respectively (*P* < 0.05). The specificity of CH-EUS in detection of portal vein involvement was relatively high compared to H-EUS (100% versus 82.6%). Therefore, The depth of invasion of biliary and ampulla cancer and vascular invasion of pancreatic and biliary cancer was demonstrated more clearly with CH-EUS compared to H-EUS. CH-EUS may improve the accuracy of preoperative T-staging of pancreaticobiliary cancer [22].

However, the results in another study that used quantitative CH-EUS for discrimination of solid pancreatic masses showed that the adenocarcinoma staging was not modified with application of CH-EUS technology compared with conventional EUS [16]. Until now it is still controversial if CH-EUS could help preoperative T-staging. Therefore, further study with more cases is needed to answer this question.

## 9. Could CH-EUS Replace EUS-Guided FNA (Avoid FNA)?

EUS-guided FNA is an accurate and safe technique to confirm the diagnosis of pancreatic cancer. The results of the EUS-FNA influenced the clinical management of the patients [23, 24]. A recent meta-analysis revealed that EUS-FNA has a high positive predictive value (99%) and a reasonable negative predictive value (64%). Because of the possible false-negative results, deciding the treatment strategy for patients with negative EUS-FNA findings still remains a challenge. 

A pilot study of 35 patients compared CH-EUS and EUS-FNA for the identification of pancreatic carcinomas, the sensitivity of CH-EUS for carcinoma identification was higher than that of EUS-FNA, and four of the five carcinomas with false-negative findings at EUS-FNA had hypoenhancement at CH-EUS [21]. Interestingly, in another study, Kitano et al. [2] reported that CH-EUS was not superior to EUS-FNA for identification of pancreatic carcinomas. However, CH-EUS revealed all ductal carcinomas with false-negative EUS-FNA results had hypoenhancement. Combining CH-EUS with EUS-FNA improved the sensitivity with which EUS-FNA identified ductal carcinomas from 92.2 to 100%. Therefore, CH-EUS before EUS-FNA complements EUS-FNA in identifying ductal carcinomas. They recommended surgical resection or pathological reevaluation by EUS-FNA of the tumor when CH-EUS revealed a hypovascular pattern in a pancreatic tumor, even if the EUS-FNA findings were negative.

Seicean et al. [16] used quantitative CH-EUS for differentiate diagnosis of solid pancreatic masses and made a conclusion that it is helpful in differentiation but cannot replace EUS-FNA. CH-EUS can be used to identify the target for EUS-guided FNA by clearly depicting the outline of the lesions [25]. Another study of 84 sessions (2.2 ± 1.2 sessions per patient, 39 patients) with a hypoechoic solid lesion in the pancreas of FNA was performed and the results revealed that in the sites with heterogeneous enhancement (*n* = 40), 8 samples (20%) were inadequate for pathological diagnosis, while malignant cells were found in 73%. In the sites with homogeneous enhancement, all samples were available for pathological diagnosis. Thus, CH-EUS may predict the pathological features of the pancreatic solid lesions. Simultaneous imaging of vascularity by CH-EUS during EUS-FNA is helpful for adequate sampling of pancreatic tumors [26].

CH-EUS, as a new technology, can visualize both parenchymal perfusion and microvasculature in pancreas without artifacts. It is useful in both differentiate diagnosis of pancreatic masses and detecting small pancreatic masses. Although it helps identify the target for EUS FNA by clearly depicting the outline and may predict the pathological features of the pancreatic lesions, but it still cannot replace EUS-FNA now.

## 10. Conclusion

Though EUS is a highly sensitive modality for detection of small lesions in the pancreas, sometimes it is hard to differentiate between diagnosis of the malignant lesion from the benign lesions. Recently developed contrast-enhanced harmonic EUS can visualize both parenchymal perfusion and microvasculature in pancreas without Doppler-related artifacts. Studies show that it is superior to conventional EUS and CT in detecting small pancreatic masses and in differentiating diagnosis of pancreatic masses. It could be applied in adequate sampling of pancreatic tumors and it may help predict the pathological features of the pancreatic lesions, but it still cannot replace EUS-FNA nowadays.

## Figures and Tables

**Table 1:** Pancreatic lesions with different vascular patterns in CH-EUS.

Lesions	Vascular patterns
Pancreatic carcinomas	Hypovascular
Neuroendocrine tumors	Hypervascular
Benign lesions	Isovascular
IPMN	Exclude avascular regions (mural nodules with enhancement)
